# Bis[2,3-bis­(thio­phen-2-yl)pyrido[3,4-*b*]pyrazine]­silver(I) perchlorate methanol disolvate

**DOI:** 10.1107/S2414314624003444

**Published:** 2024-04-26

**Authors:** Guy Crundwell, Rachel A. Christiana, Paul Ouellette, Andrew F. Giorgetti

**Affiliations:** a Central Connecticut State University, Department of Chemistry & Biochemistry, 1619 Stanley Street, New Britain, CT 06053, USA; Purdue University, USA

**Keywords:** crystal structure, pyrido­pyrazine, silver, thienyl disodered

## Abstract

The title compound, [Ag(C_15_H_9_N_3_S_2_)_2_]ClO_4_·2CH_3_OH, is monoclinic. The Ag^I^ atom is coordinated by pyrido N atoms and is two-coordinate; however, the Ag^I^ atom has nearby O atoms that can be assumed to be weakly bonded – one from the perchlorate anion and one from the methanol solvate molecule. One of the thienyl groups on a 2,3-bis­(thio­phen-2-yl)pyrido[3,4-*b*]pyrazine is flipped disordered and was refined to occupancies of 68.4 (6) and 31.6 (6)%.

## Abstract 2

The structure of bis­[2,3-bis­(thio­phen-2-yl)pyrido[3,4-*b*]pyrazine]­silver(I) perchlorate methanol disolvate is monoclinic. The Ag atom coordinates pyrido N atoms and is two-coordinate; however, the Ag atom has nearby O atoms that can be assumed to be weakly bonding – one from the perchlorate anion and one from the methanol solvate. One of the thienyl groups of a 2,3-bis­(thio­phen-2-yl)pyrido[3,4-*b*]pyrazine is flipped disordered and was refined to occupancies of 68.4 (6) and 31.6 (6)%.

## Graphical abstract

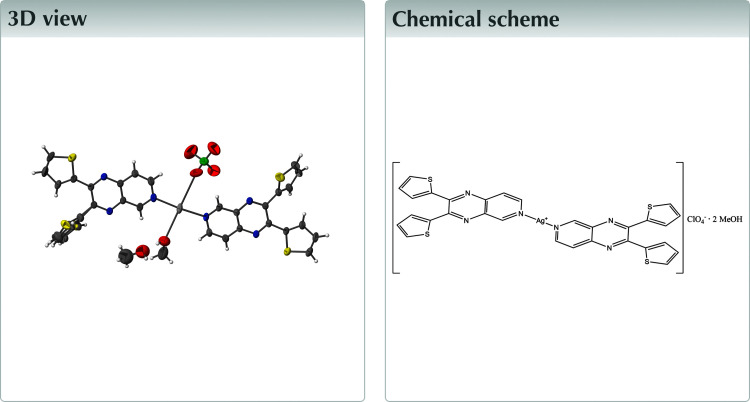

## Structure description

Crystal structures of di­aryl­pyrido[2,3-*b*]pyrazines are well known. For example, the authors have published the crystal structures of 2,3-bis­(thio­phen-2-yl)pyrido[2,3-*b*]pyrazine and 7-bromo-2,3-bis­(thio­phen-2-yl)pyrido[2,3-*b*]pyrazine (Popek & Crundwell, 2019 ▸). By comparison, only one structure of a pyrido[3,4-*b*]pyrazine has been published to date, namely, 2,3-di­phenyl­pyrido[3,4-*b*]pyrazine (Chan & Chang, 2016 ▸). This article is the first single-crystal XRD study of dithienylpyrido[3,4-*b*]pyrazine, as well as the first *bis* complex of this ligand with a transition metal.

**Figure 1:**
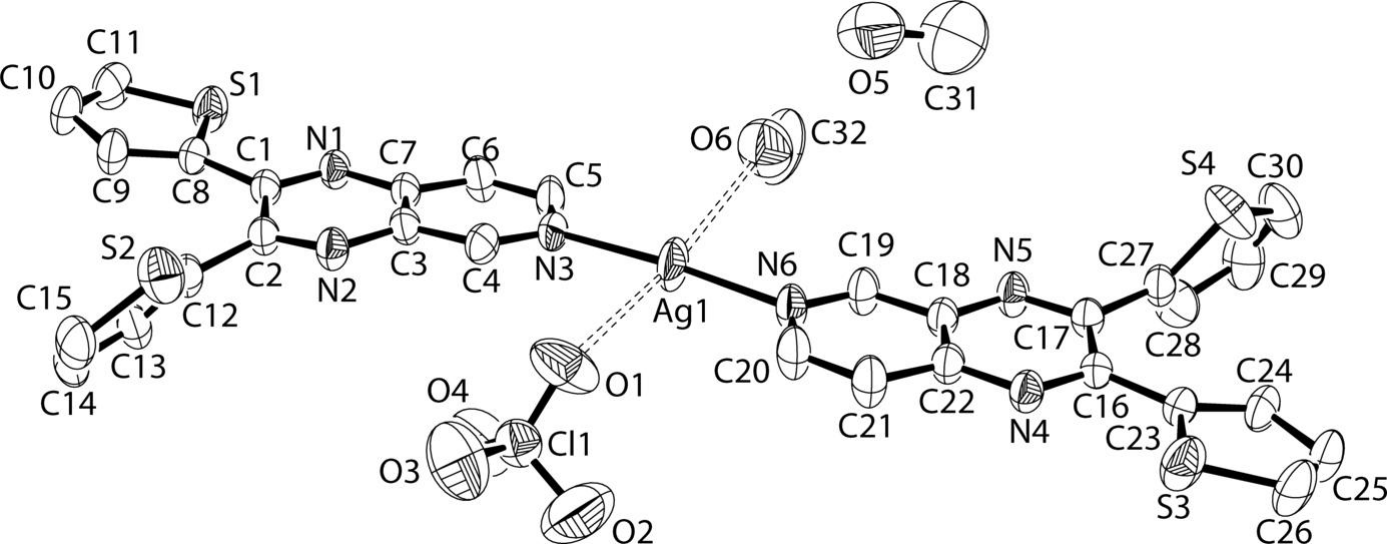
A view of the title compound. Displacement ellipsoids are drawn at the 50% probability level. The dotted bonds illustrate the nearby O atoms from a methanol and from the counter-anion. All H atoms have been omitted, as has the minor component of the disordered thienyl ring.

The *bis* complex with silver utilizes the pyrido N atom in the pyrido[3,4-*b*]pyrazine to make a nearly linear, nearly flat silver(I) complex where the N—Ag—N angle is 175.25 (14)°. This is inter­esting compared to *bis* complexes with 2,3-di­aryl­quinoxalines which have no choice but to bond to metals using the quinoxaline N atoms, which are more sterically hindered due to their close proximity to the aryl groups on neighboring C atoms. Additionally, the Ag^I^ atom is weakly coordinated by two O atoms – a methanol O atom (O6, Fig. 1 ▸) at 2.782 (4) Å and a perchlorate O atom (O1) at 3.079 (5) Å, thereby mimicking a four-coordinate square-planar environment (Table 1 ▸).

Like many *bis* 2,3-dithienylquinoxaline complexes with metals where one thienyl ring is nearly coplanar with the quinoxaline ring (Crundwell & Ellis, 2023 ▸), here also one thienyl ring in each ligand is nearly planar with the main pyrido­pyrazine moiety. Based on least-squares-plane calculations, the thienyl rings containing S1 and S3 (Fig. 1 ▸) make angles of 11.2 (2) and 4.35 (11)°, respectively, with respect to the least-squares-plane determinations of the pyrido­py­ra­zine atoms. By comparison, the thienyl rings containing the S2 and S4 atoms make angles of 50.36 (11) and 64.5 (5)°. Also typical for these thienyl rings are flip disorders. The thienyl ring containing the S4 atom exhibits a flip disorder of 68.4 (6)/31.6 (6)%.

Finally, there are several hydrogen bonds in the structure (Table 2 ▸). The most significant hydrogen bonds involve the hydroxy groups on the two methanol solvent molecules. The methanol O atom (O6, Fig. 1 ▸) that weakly coordinates to the Ag^I^ atom also has a H atom that is hydrogen bonded to the neighboring methanol (O5), which, in turn, particpates in a hydrogen bond by donating its H atom to a symmetry-related perchlorate anion.

## Synthesis and crystallization

Silver perchlorate was used as received from Fisher chemicals. 2,3-Bis(thio­phen-2-yl)pyrido[3,4-*b*]pyrazine was synthesized by the acid-catalyzed condensation reaction between 2,2′-thenil and pyridine-3,4-di­amine (Lassagne *et al.*, 2015 ▸) and was purified by column chromatography before use.

A 30 ml methanol solution of 148 mg (0.50 mmol) of pyrido­pyrazine was stirred and warmed until the ligand dissolved. A 5 ml solution of 52 mg (0.25 mmol) of silver perchlorate in methanol was added to the former solution. The resulting mixture was removed from heat and transferred into test tubes that were individually placed into amber vials. The amber vials were loosely capped and were placed in a drawer to remove them from ambient room lighting. Diffraction-quality pale-yellow plates formed *via* slow evaporation of the solvent within 48 h. Crystals were harvested from the evaporating solutions. Crystals slowly desolvated upon standing in open air and were thus covered in paraffin oil for data collection.

## Refinement

Crystal data, data collection and structure refinement details are summarized in Table 3 ▸.

A ring-flip disorder of 68.4 (6) to 31.6 (6)% was determined for one of the ligand thienyl rings. This disorder was treated by a FLAT restraint to the flipped component atoms of the thienyl ring along with SADI and SIMU restraints to control bond lengths and displacement parameters, respectively. The displacement parameter of the C atom that connects the thienyl ring to the pyrido­pyrazine ring was constrained to be idenical in both flipped orientations using an EADP constraint.

Finally, an H atom on a methanol solvent molecule was restrained such that it made a hydrogen bond with a neighboring methanol.

## Supplementary Material

Crystal structure: contains datablock(s) I. DOI: 10.1107/S2414314624003444/zl4071sup1.cif

Structure factors: contains datablock(s) I. DOI: 10.1107/S2414314624003444/zl4071Isup2.hkl

CCDC reference: 2349141

Additional supporting information:  crystallographic information; 3D view; checkCIF report

## Figures and Tables

**Table 1:** Selected geometric parameters (Å, °)

Ag1—N3	2.170 (3)	Ag1—O1	3.079 (5)
Ag1—N6	2.179 (3)	Ag1—O6	2.782 (4)
			
N3—Ag1—N6	175.24 (14)		

**Table 2:** Hydrogen-bond geometry (Å, °)

*D*—H⋯*A*	*D*—H	H⋯*A*	*D*⋯*A*	*D*—H⋯*A*
C6—H6⋯S4^i^	0.93	2.92	3.665 (6)	138
C9—H9⋯O2^ii^	0.93	2.64	3.465 (8)	149
C11—H11⋯O5^i^	0.93	2.65	3.520 (8)	156
C14—H14⋯N5^iii^	0.93	2.69	3.393 (5)	133
C24—H24⋯S4	0.93	2.77	3.321 (6)	119
C24—H24⋯S4*B*	0.93	3.00	3.774 (17)	141
C29—H29⋯O4^iv^	0.93	2.49	3.330 (12)	150
C30—H30⋯Cl1^v^	0.93	2.96	3.738 (10)	142
C30—H30⋯O1^v^	0.93	2.40	3.312 (10)	166
C28*B*—H28*B*⋯S1^vi^	0.93	2.93	3.67 (2)	138
C29*B*—H29*B*⋯O2^v^	0.93	2.31	3.21 (2)	162
O6—H6*A*⋯O5	0.82	1.99	2.805 (8)	175
O5—H5*A*⋯O3^vii^	0.82	2.11	2.889 (8)	158

Symmetry codes: (i) 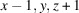 ; (ii) 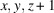 ; (iii) 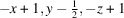 ; (iv) 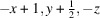 ; (v) 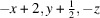 ; (vi) 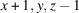 ; (vii) 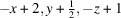 .

**Table 3:** Experimental details

Crystal data	
Chemical formula	[Ag(C_15_H_9_N_3_S_2_)_2_]ClO_4_·2CH_4_O
*M*_r_	862.15
Crystal system, space group	Monoclinic, *P*2_1_
Temperature (K)	293
*a*, *b*, *c* (Å)	8.3455 (3), 19.2331 (6), 11.0134 (3)
β (°)	102.491 (3)
*V* (Å^3^)	1725.93 (10)
*Z*	2
Radiation type	Mo *K*α
μ (mm^−1^)	0.96
Crystal size (mm)	0.38 × 0.37 × 0.11

Data collection	
Diffractometer	Xcalibur Sapphire3
Absorption correction	Multi-scan (*CrysAlis PRO*; Rigaku OD, 2019 ▸)
*T*_min_, *T*_max_	0.851, 1.000
No. of measured, independent and observed [*I* > 2σ(*I*)] reflections	45050, 12591, 8576
*R*_int_	0.039
(sin θ/λ)_max_ (Å^−1^)	0.780

Refinement	
*R*[*F* ^2^ > 2σ(*F* ^2^)], *wR*(*F* ^2^), *S*	0.041, 0.095, 1.01
No. of reflections	12591
No. of parameters	495
No. of restraints	185
H-atom treatment	H-atom parameters constrained
Δρ_max_, Δρ_min_ (e Å^−3^)	0.45, −0.28
Absolute structure	Flack *x* determined using 3323 quotients [(*I* ^+^)−(*I* ^−^)]/[(*I* ^+^)+(*I* ^−^)] (Parsons *et al.*, 2013 ▸)
Absolute structure parameter	−0.044 (8)

Computer programs: *CrysAlis PRO* (Rigaku OD, 2019 ▸), *SHELXT* (Sheldrick, 2015*b* ▸), *SHELXL* (Sheldrick, 2015*a* ▸), *ORTEP for Windows* (Farrugia, 2012 ▸) and *OLEX2* (Dolomanov *et al.*, 2009 ▸).

## full crystallographic data

### Crystal data

**Table:**

[Ag(C_15_H_9_N_3_S_2_)_2_]ClO_4_·2CH_4_O	*F*(000) = 872
*M**_r_* = 862.15	*D*_x_ = 1.659 Mg m^−^^3^
Monoclinic, *P*2_1_	Mo *K*α radiation, λ = 0.71073 Å
*a* = 8.3455 (3) Å	Cell parameters from 10547 reflections
*b* = 19.2331 (6) Å	θ = 3.1–31.8°
*c* = 11.0134 (3) Å	µ = 0.96 mm^−^^1^
β = 102.491 (3)°	*T* = 293 K
*V* = 1725.93 (10) Å^3^	Plate, yellow
*Z* = 2	0.38 × 0.37 × 0.11 mm

### Data collection

**Table:**

Xcalibur Sapphire3 diffractometer	12591 independent reflections
Radiation source: fine-focus sealed X-ray tube, Enhance (Mo) X-ray Source	8576 reflections with *I* > 2σ(*I*)
Graphite monochromator	*R*_int_ = 0.039
Detector resolution: 16.1790 pixels mm^-1^	θ_max_ = 33.7°, θ_min_ = 3.0°
ω scans	*h* = −12→12
Absorption correction: multi-scan (CrysAlis PRO; Rigaku OD, 2019)	*k* = −29→29
*T*_min_ = 0.851, *T*_max_ = 1.000	*l* = −16→16
45050 measured reflections	

### Refinement

**Table:**

Refinement on *F*^2^	Hydrogen site location: inferred from neighbouring sites
Least-squares matrix: full	H-atom parameters constrained
*R*[*F*^2^ > 2σ(*F*^2^)] = 0.041	*w* = 1/[σ^2^(*F*_o_^2^) + (0.0407*P*)^2^ + 0.1482*P*] where *P* = (*F*_o_^2^ + 2*F*_c_^2^)/3
*wR*(*F*^2^) = 0.095	(Δ/σ)_max_ < 0.001
*S* = 1.01	Δρ_max_ = 0.45 e Å^−^^3^
12591 reflections	Δρ_min_ = −0.28 e Å^−^^3^
495 parameters	Absolute structure: Flack *x* determined using 3323 quotients [(*I*^+^)-(*I*^-^)]/[(*I*^+^)+(*I*^-^)] (Parsons *et al.*, 2013)
185 restraints	Absolute structure parameter: −0.044 (8)

### Special details

**Table:**

Geometry. All esds (except the esd in the dihedral angle between two l.s. planes) are estimated using the full covariance matrix. The cell esds are taken into account individually in the estimation of esds in distances, angles and torsion angles; correlations between esds in cell parameters are only used when they are defined by crystal symmetry. An approximate (isotropic) treatment of cell esds is used for estimating esds involving l.s. planes.
Refinement. H atoms on *sp*^2^ and *sp*^3^ C atom were placed at calculated positions with C—H distances of 0.93 and 0.96 Å and were included in the refinement in riding-motion approximation, with *U*_iso_(H) = 1.2*U*_eq_ or 1.5*U*_eq_ of the carrier atom, respectively. Hydroxy-group H atoms were also placed at calculated positions with an O—H distance of 0.82 Å and were included in the refinement in riding-motion approximation, with 1.5*U*_eq_ of the carrier atom.

### Fractional atomic coordinates and isotropic or equivalent isotropic displacement parameters (Å^2^)

**Table:**

	*x*	*y*	*z*	*U*_iso_*/*U*_eq_	Occ. (<1)
Ag1	0.70442 (4)	0.12456 (2)	0.38256 (3)	0.05936 (11)	
N1	0.4283 (4)	0.14950 (16)	0.8719 (3)	0.0373 (6)	
N2	0.5361 (4)	0.02155 (15)	0.7974 (3)	0.0397 (7)	
N3	0.6104 (4)	0.1316 (2)	0.5510 (2)	0.0421 (7)	
N4	0.9634 (4)	0.07609 (16)	−0.1102 (3)	0.0383 (7)	
N5	0.8781 (4)	0.20922 (16)	−0.0416 (3)	0.0405 (7)	
N6	0.7905 (4)	0.10874 (17)	0.2114 (3)	0.0455 (8)	
C1	0.4227 (4)	0.09252 (19)	0.9368 (3)	0.0345 (7)	
C2	0.4642 (4)	0.02578 (18)	0.8917 (3)	0.0350 (7)	
C3	0.5467 (4)	0.08089 (19)	0.7322 (3)	0.0356 (7)	
C4	0.6119 (5)	0.0784 (2)	0.6252 (3)	0.0409 (8)	
H4	0.658463	0.037029	0.606024	0.049*	
C5	0.5462 (6)	0.1931 (2)	0.5815 (4)	0.0486 (10)	
H5	0.545322	0.231182	0.529383	0.058*	
C6	0.4834 (6)	0.2006 (2)	0.6851 (4)	0.0484 (10)	
H6	0.440309	0.243127	0.702726	0.058*	
C7	0.4843 (5)	0.14404 (17)	0.7647 (3)	0.0352 (7)	
C8	0.3768 (4)	0.10390 (18)	1.0565 (3)	0.0360 (8)	
C9	0.3805 (5)	0.0610 (2)	1.1561 (4)	0.0465 (9)	
H9	0.409226	0.014285	1.157075	0.056*	
C10	0.3359 (6)	0.0953 (3)	1.2574 (4)	0.0538 (11)	
H10	0.332455	0.073679	1.332314	0.065*	
C11	0.2991 (7)	0.1626 (3)	1.2335 (4)	0.0579 (12)	
H11	0.266068	0.192752	1.289439	0.070*	
S1	0.32009 (16)	0.18639 (6)	1.08882 (10)	0.0512 (3)	
C12	0.4263 (5)	−0.04052 (19)	0.9440 (3)	0.0415 (8)	
C13	0.2780 (6)	−0.0623 (2)	0.9634 (4)	0.0476 (9)	
H13	0.185986	−0.033838	0.953629	0.057*	
C14	0.2801 (7)	−0.1323 (2)	0.9996 (4)	0.0577 (12)	
H14	0.189238	−0.155270	1.016382	0.069*	
C15	0.4267 (7)	−0.1627 (2)	1.0074 (5)	0.0631 (13)	
H15	0.449611	−0.208621	1.031537	0.076*	
S2	0.56527 (17)	−0.10719 (6)	0.96782 (13)	0.0613 (3)	
C16	0.9753 (4)	0.1309 (2)	−0.1800 (3)	0.0331 (6)	
C17	0.9372 (4)	0.19958 (18)	−0.1415 (3)	0.0349 (7)	
C18	0.8609 (5)	0.15199 (19)	0.0273 (3)	0.0359 (8)	
C19	0.8032 (5)	0.1610 (2)	0.1367 (4)	0.0447 (9)	
H19	0.773010	0.205273	0.157263	0.054*	
C20	0.8349 (6)	0.0447 (2)	0.1796 (4)	0.0532 (11)	
H20	0.824813	0.007681	0.231893	0.064*	
C21	0.8936 (6)	0.0311 (2)	0.0755 (4)	0.0503 (10)	
H21	0.923412	−0.013854	0.058245	0.060*	
C22	0.9079 (5)	0.0861 (2)	−0.0047 (3)	0.0369 (7)	
C23	1.0254 (4)	0.1167 (2)	−0.2965 (3)	0.0367 (7)	
C24	1.0302 (5)	0.1588 (2)	−0.3989 (3)	0.0448 (9)	
H24	1.002324	0.205685	−0.404821	0.054*	
C25	1.0833 (5)	0.1202 (3)	−0.4929 (4)	0.0587 (11)	
H25	1.092595	0.139355	−0.568554	0.070*	
C26	1.1189 (7)	0.0532 (3)	−0.4622 (4)	0.0639 (13)	
H26	1.157432	0.021711	−0.513264	0.077*	
S3	1.08557 (18)	0.03321 (6)	−0.32037 (11)	0.0599 (3)	
C27	0.9611 (16)	0.2629 (6)	−0.2120 (14)	0.0367 (17)	0.684 (6)
C28	0.842 (2)	0.3113 (12)	−0.258 (2)	0.049 (3)	0.684 (6)
H28	0.731684	0.307225	−0.255469	0.058*	0.684 (6)
C29	0.9107 (12)	0.3688 (6)	−0.3118 (12)	0.062 (2)	0.684 (6)
H29	0.847376	0.404312	−0.355070	0.074*	0.684 (6)
C30	1.0712 (13)	0.3668 (5)	−0.2944 (10)	0.061 (3)	0.684 (6)
H30	1.133736	0.401005	−0.321978	0.073*	0.684 (6)
S4	1.1517 (4)	0.2942 (2)	−0.2149 (3)	0.0632 (9)	0.684 (6)
C27B	0.976 (3)	0.2650 (14)	−0.194 (3)	0.0367 (17)	0.316 (6)
C28B	1.132 (3)	0.2911 (15)	−0.184 (2)	0.051 (5)	0.316 (6)
H28B	1.227018	0.272075	−0.135305	0.061*	0.316 (6)
C29B	1.124 (2)	0.3524 (11)	−0.259 (2)	0.059 (5)	0.316 (6)
H29B	1.217078	0.376431	−0.269730	0.071*	0.316 (6)
C30B	0.975 (2)	0.3713 (13)	−0.311 (3)	0.064 (6)	0.316 (6)
H30B	0.949664	0.411530	−0.358317	0.076*	0.316 (6)
S4B	0.8309 (15)	0.3144 (8)	−0.2838 (14)	0.062 (3)	0.316 (6)
Cl1	0.56109 (14)	−0.07882 (6)	0.41362 (11)	0.0552 (3)	
O1	0.6944 (5)	−0.0331 (2)	0.4288 (5)	0.0981 (16)	
O2	0.5177 (8)	−0.0979 (4)	0.2865 (5)	0.131 (2)	
O3	0.6003 (7)	−0.1420 (3)	0.4721 (6)	0.1162 (17)	
O4	0.4275 (5)	−0.0473 (2)	0.4526 (5)	0.0983 (15)	
O6	0.7948 (6)	0.2632 (2)	0.3707 (5)	0.0914 (13)	
H6A	0.887327	0.273455	0.362682	0.137*	
C32	0.6934 (10)	0.3214 (4)	0.3467 (7)	0.107 (2)	
H32A	0.732501	0.351764	0.290270	0.161*	
H32B	0.694406	0.345596	0.423134	0.161*	
H32C	0.583375	0.306980	0.310247	0.161*	
O5	1.1150 (7)	0.3021 (3)	0.3591 (5)	0.1150 (17)	
H5A	1.178122	0.318490	0.419943	0.172*	
C31	1.1213 (12)	0.3419 (6)	0.2541 (10)	0.133 (3)	
H31A	1.075958	0.315668	0.180458	0.200*	
H31B	1.233285	0.353542	0.254607	0.200*	
H31C	1.058898	0.383737	0.254938	0.200*	

### Atomic displacement parameters (Å^2^)

**Table:**

	*U*^11^	*U*^22^	*U*^33^	*U*^12^	*U*^13^	*U*^23^
Ag1	0.0787 (2)	0.0712 (2)	0.03931 (14)	−0.0011 (2)	0.03710 (14)	0.00273 (17)
N1	0.0452 (16)	0.0377 (15)	0.0334 (15)	−0.0010 (12)	0.0185 (13)	0.0006 (12)
N2	0.0520 (18)	0.0362 (15)	0.0359 (15)	0.0013 (14)	0.0203 (13)	0.0022 (12)
N3	0.0527 (16)	0.0490 (18)	0.0301 (13)	−0.0117 (17)	0.0209 (12)	−0.0033 (16)
N4	0.0506 (18)	0.0346 (15)	0.0346 (16)	0.0000 (13)	0.0198 (13)	−0.0012 (12)
N5	0.0541 (19)	0.0346 (15)	0.0395 (16)	0.0052 (14)	0.0253 (15)	0.0021 (12)
N6	0.0564 (19)	0.048 (2)	0.0394 (16)	−0.0015 (15)	0.0265 (14)	0.0044 (13)
C1	0.0371 (17)	0.0383 (17)	0.0312 (16)	−0.0020 (14)	0.0142 (14)	0.0018 (14)
C2	0.0413 (18)	0.0339 (17)	0.0328 (16)	−0.0008 (14)	0.0145 (14)	0.0031 (13)
C3	0.0419 (19)	0.0345 (17)	0.0338 (17)	−0.0047 (15)	0.0159 (15)	0.0011 (14)
C4	0.049 (2)	0.045 (2)	0.0336 (18)	−0.0026 (17)	0.0208 (16)	−0.0062 (15)
C5	0.070 (3)	0.045 (2)	0.038 (2)	−0.005 (2)	0.0279 (19)	0.0064 (17)
C6	0.066 (3)	0.038 (2)	0.050 (2)	−0.0016 (19)	0.031 (2)	0.0035 (17)
C7	0.0433 (18)	0.0352 (19)	0.0311 (15)	−0.0051 (14)	0.0169 (13)	−0.0004 (13)
C8	0.0413 (18)	0.042 (2)	0.0284 (15)	−0.0024 (14)	0.0148 (13)	−0.0002 (12)
C9	0.064 (3)	0.046 (2)	0.0344 (19)	0.0014 (19)	0.0212 (18)	0.0059 (16)
C10	0.070 (3)	0.067 (3)	0.0303 (19)	0.002 (2)	0.0242 (19)	0.0060 (18)
C11	0.076 (3)	0.065 (3)	0.040 (2)	−0.001 (2)	0.030 (2)	−0.011 (2)
S1	0.0749 (7)	0.0455 (5)	0.0399 (5)	0.0041 (5)	0.0270 (5)	−0.0016 (4)
C12	0.056 (2)	0.0373 (19)	0.0351 (18)	−0.0012 (16)	0.0192 (16)	0.0057 (15)
C13	0.059 (2)	0.041 (2)	0.048 (2)	−0.0051 (18)	0.0227 (19)	0.0075 (17)
C14	0.083 (3)	0.046 (2)	0.048 (2)	−0.019 (2)	0.023 (2)	0.0058 (19)
C15	0.099 (4)	0.037 (2)	0.056 (3)	0.000 (2)	0.022 (3)	0.012 (2)
S2	0.0724 (8)	0.0425 (5)	0.0732 (8)	0.0112 (5)	0.0251 (6)	0.0113 (5)
C16	0.0355 (15)	0.0359 (17)	0.0301 (14)	−0.0043 (16)	0.0120 (11)	−0.0010 (16)
C17	0.0393 (19)	0.0349 (18)	0.0334 (17)	0.0011 (14)	0.0144 (14)	0.0019 (14)
C18	0.0426 (19)	0.0373 (17)	0.0321 (17)	−0.0013 (14)	0.0175 (14)	−0.0005 (13)
C19	0.057 (2)	0.045 (2)	0.041 (2)	0.0036 (18)	0.0283 (18)	0.0023 (16)
C20	0.075 (3)	0.046 (2)	0.047 (2)	0.003 (2)	0.031 (2)	0.0122 (18)
C21	0.077 (3)	0.0371 (19)	0.045 (2)	0.003 (2)	0.032 (2)	0.0048 (17)
C22	0.0447 (19)	0.0365 (17)	0.0329 (17)	−0.0003 (16)	0.0157 (15)	0.0010 (14)
C23	0.0394 (16)	0.0424 (19)	0.0309 (14)	−0.0010 (16)	0.0131 (12)	−0.0037 (15)
C24	0.050 (2)	0.057 (2)	0.0316 (18)	0.0035 (18)	0.0194 (16)	−0.0040 (16)
C25	0.069 (2)	0.079 (3)	0.0351 (17)	−0.005 (3)	0.0272 (17)	−0.005 (3)
C26	0.085 (3)	0.074 (3)	0.041 (2)	0.008 (3)	0.033 (2)	−0.015 (2)
S3	0.0897 (9)	0.0492 (6)	0.0473 (6)	0.0080 (6)	0.0294 (6)	−0.0066 (5)
C27	0.049 (3)	0.0351 (18)	0.030 (5)	−0.0042 (18)	0.018 (3)	−0.001 (2)
C28	0.057 (6)	0.047 (6)	0.047 (7)	−0.002 (5)	0.023 (5)	0.010 (4)
C29	0.072 (6)	0.049 (4)	0.068 (5)	0.010 (5)	0.024 (5)	0.018 (4)
C30	0.069 (7)	0.042 (4)	0.080 (7)	−0.011 (5)	0.033 (6)	0.011 (4)
S4	0.0515 (12)	0.0482 (13)	0.098 (2)	−0.0056 (9)	0.0335 (12)	0.0089 (14)
C27B	0.049 (3)	0.0351 (18)	0.030 (5)	−0.0042 (18)	0.018 (3)	−0.001 (2)
C28B	0.061 (10)	0.036 (7)	0.063 (9)	−0.006 (7)	0.029 (7)	0.022 (7)
C29B	0.057 (9)	0.057 (9)	0.065 (9)	−0.006 (7)	0.013 (7)	0.019 (7)
C30B	0.060 (12)	0.054 (8)	0.077 (9)	−0.003 (11)	0.013 (11)	0.032 (7)
S4B	0.073 (4)	0.049 (3)	0.061 (6)	0.003 (3)	0.009 (3)	0.017 (3)
Cl1	0.0634 (6)	0.0444 (5)	0.0612 (6)	0.0008 (5)	0.0208 (5)	−0.0028 (5)
O1	0.093 (3)	0.072 (3)	0.150 (4)	−0.031 (2)	0.074 (3)	−0.054 (3)
O2	0.141 (4)	0.165 (5)	0.079 (3)	−0.014 (4)	0.010 (3)	−0.039 (4)
O3	0.125 (4)	0.089 (3)	0.142 (5)	0.008 (3)	0.046 (3)	0.037 (3)
O4	0.077 (3)	0.083 (3)	0.150 (4)	0.012 (2)	0.058 (3)	−0.007 (3)
O6	0.118 (3)	0.063 (2)	0.099 (3)	−0.007 (2)	0.036 (3)	−0.003 (2)
C32	0.150 (7)	0.106 (5)	0.080 (4)	0.044 (5)	0.053 (4)	0.017 (4)
O5	0.113 (4)	0.122 (4)	0.103 (4)	−0.025 (3)	0.010 (3)	−0.018 (3)
C31	0.132 (7)	0.133 (8)	0.131 (8)	0.022 (6)	0.019 (6)	0.017 (6)

### Geometric parameters (Å, º)

**Table:**

Ag1—N3	2.170 (3)	C18—C19	1.402 (5)
Ag1—N6	2.179 (3)	C18—C22	1.394 (5)
Ag1—O1	3.079 (5)	C19—H19	0.9300
Ag1—O6	2.782 (4)	C20—H20	0.9300
N1—C1	1.315 (4)	C20—C21	1.366 (5)
N1—C7	1.365 (4)	C21—H21	0.9300
N2—C2	1.309 (4)	C21—C22	1.401 (5)
N2—C3	1.362 (4)	C23—C24	1.396 (5)
N3—C4	1.308 (5)	C23—S3	1.720 (4)
N3—C5	1.369 (6)	C24—H24	0.9300
N4—C16	1.321 (5)	C24—C25	1.420 (5)
N4—C22	1.354 (5)	C25—H25	0.9300
N5—C17	1.313 (4)	C25—C26	1.348 (8)
N5—C18	1.361 (5)	C26—H26	0.9300
N6—C19	1.317 (5)	C26—S3	1.690 (5)
N6—C20	1.354 (5)	C27—C28	1.379 (18)
C1—C2	1.445 (5)	C27—S4	1.708 (11)
C1—C8	1.467 (5)	C28—H28	0.9300
C2—C12	1.462 (5)	C28—C29	1.429 (18)
C3—C4	1.402 (5)	C29—H29	0.9300
C3—C7	1.398 (5)	C29—C30	1.312 (11)
C4—H4	0.9300	C30—H30	0.9300
C5—H5	0.9300	C30—S4	1.707 (10)
C5—C6	1.363 (5)	C27B—C28B	1.38 (2)
C6—H6	0.9300	C27B—S4B	1.68 (2)
C6—C7	1.396 (5)	C28B—H28B	0.9300
C8—C9	1.367 (5)	C28B—C29B	1.44 (2)
C8—S1	1.715 (4)	C29B—H29B	0.9300
C9—H9	0.9300	C29B—C30B	1.309 (19)
C9—C10	1.413 (6)	C30B—H30B	0.9300
C10—H10	0.9300	C30B—S4B	1.698 (18)
C10—C11	1.344 (7)	Cl1—O1	1.399 (4)
C11—H11	0.9300	Cl1—O2	1.417 (5)
C11—S1	1.703 (5)	Cl1—O3	1.380 (5)
C12—C13	1.365 (6)	Cl1—O4	1.415 (4)
C12—S2	1.711 (4)	O6—H6A	0.8200
C13—H13	0.9300	O6—C32	1.394 (8)
C13—C14	1.404 (6)	C32—H32A	0.9600
C14—H14	0.9300	C32—H32B	0.9600
C14—C15	1.342 (7)	C32—H32C	0.9600
C15—H15	0.9300	O5—H5A	0.8200
C15—S2	1.699 (5)	O5—C31	1.398 (11)
C16—C17	1.443 (5)	C31—H31A	0.9600
C16—C23	1.458 (4)	C31—H31B	0.9600
C17—C27	1.480 (8)	C31—H31C	0.9600
C17—C27B	1.451 (16)		
			
N3—Ag1—N6	175.24 (14)	N6—C19—H19	118.9
C1—N1—C7	117.7 (3)	C18—C19—H19	118.9
C2—N2—C3	117.3 (3)	N6—C20—H20	118.0
C4—N3—Ag1	121.9 (3)	N6—C20—C21	123.9 (4)
C4—N3—C5	118.3 (3)	C21—C20—H20	118.0
C5—N3—Ag1	119.8 (3)	C20—C21—H21	120.7
C16—N4—C22	117.9 (3)	C20—C21—C22	118.7 (4)
C17—N5—C18	117.2 (3)	C22—C21—H21	120.7
C19—N6—Ag1	121.3 (3)	N4—C22—C18	120.9 (3)
C19—N6—C20	118.0 (3)	N4—C22—C21	121.7 (3)
C20—N6—Ag1	120.7 (2)	C18—C22—C21	117.4 (3)
N1—C1—C2	121.0 (3)	C16—C23—S3	117.2 (3)
N1—C1—C8	114.3 (3)	C24—C23—C16	131.8 (4)
C2—C1—C8	124.8 (3)	C24—C23—S3	110.9 (3)
N2—C2—C1	120.8 (3)	C23—C24—H24	124.6
N2—C2—C12	115.7 (3)	C23—C24—C25	110.7 (4)
C1—C2—C12	123.4 (3)	C25—C24—H24	124.6
N2—C3—C4	119.8 (3)	C24—C25—H25	123.1
N2—C3—C7	121.5 (3)	C26—C25—C24	113.8 (4)
C7—C3—C4	118.6 (3)	C26—C25—H25	123.1
N3—C4—C3	123.1 (4)	C25—C26—H26	123.9
N3—C4—H4	118.5	C25—C26—S3	112.2 (3)
C3—C4—H4	118.5	S3—C26—H26	123.9
N3—C5—H5	118.7	C26—S3—C23	92.3 (2)
C6—C5—N3	122.6 (4)	C17—C27—S4	122.1 (8)
C6—C5—H5	118.7	C28—C27—C17	125.6 (12)
C5—C6—H6	120.3	C28—C27—S4	110.8 (9)
C5—C6—C7	119.4 (4)	C27—C28—H28	124.7
C7—C6—H6	120.3	C27—C28—C29	110.7 (13)
N1—C7—C3	120.3 (3)	C29—C28—H28	124.7
N1—C7—C6	121.8 (3)	C28—C29—H29	123.0
C6—C7—C3	118.0 (3)	C30—C29—C28	114.0 (12)
C1—C8—S1	117.5 (3)	C30—C29—H29	123.0
C9—C8—C1	131.8 (3)	C29—C30—H30	124.0
C9—C8—S1	110.5 (3)	C29—C30—S4	112.0 (9)
C8—C9—H9	123.6	S4—C30—H30	124.0
C8—C9—C10	112.8 (4)	C27—S4—C30	92.0 (5)
C10—C9—H9	123.6	C17—C27B—S4B	122.3 (17)
C9—C10—H10	123.7	C28B—C27B—C17	125 (2)
C11—C10—C9	112.5 (4)	C28B—C27B—S4B	113.0 (13)
C11—C10—H10	123.7	C27B—C28B—H28B	125.4
C10—C11—H11	123.9	C27B—C28B—C29B	109.1 (18)
C10—C11—S1	112.1 (4)	C29B—C28B—H28B	125.4
S1—C11—H11	123.9	C28B—C29B—H29B	123.2
C11—S1—C8	92.0 (2)	C30B—C29B—C28B	113.6 (19)
C2—C12—S2	121.1 (3)	C30B—C29B—H29B	123.2
C13—C12—C2	127.5 (4)	C29B—C30B—H30B	123.6
C13—C12—S2	110.7 (3)	C29B—C30B—S4B	112.8 (16)
C12—C13—H13	123.8	S4B—C30B—H30B	123.6
C12—C13—C14	112.5 (4)	C27B—S4B—C30B	91.3 (11)
C14—C13—H13	123.8	O1—Cl1—O2	107.9 (3)
C13—C14—H14	123.5	O1—Cl1—O4	110.6 (3)
C15—C14—C13	113.0 (4)	O3—Cl1—O1	113.3 (3)
C15—C14—H14	123.5	O3—Cl1—O2	102.6 (4)
C14—C15—H15	124.1	O3—Cl1—O4	111.0 (3)
C14—C15—S2	111.8 (3)	O4—Cl1—O2	111.2 (4)
S2—C15—H15	124.1	C32—O6—H6A	109.5
C15—S2—C12	91.9 (2)	O6—C32—H32A	109.5
N4—C16—C17	120.7 (3)	O6—C32—H32B	109.5
N4—C16—C23	115.8 (4)	O6—C32—H32C	109.5
C17—C16—C23	123.5 (3)	H32A—C32—H32B	109.5
N5—C17—C16	121.5 (3)	H32A—C32—H32C	109.5
N5—C17—C27	116.2 (8)	H32B—C32—H32C	109.5
N5—C17—C27B	111.7 (17)	C31—O5—H5A	109.5
C16—C17—C27	122.4 (7)	O5—C31—H31A	109.5
C16—C17—C27B	126.4 (17)	O5—C31—H31B	109.5
N5—C18—C19	118.4 (3)	O5—C31—H31C	109.5
N5—C18—C22	121.6 (3)	H31A—C31—H31B	109.5
C22—C18—C19	119.8 (3)	H31A—C31—H31C	109.5
N6—C19—C18	122.1 (4)	H31B—C31—H31C	109.5
			
Ag1—N3—C4—C3	−177.7 (3)	C9—C10—C11—S1	−0.8 (6)
Ag1—N3—C5—C6	179.2 (4)	C10—C11—S1—C8	0.9 (5)
Ag1—N6—C19—C18	178.9 (3)	S1—C8—C9—C10	0.5 (5)
Ag1—N6—C20—C21	−178.4 (3)	C12—C13—C14—C15	0.0 (6)
N1—C1—C2—N2	12.4 (5)	C13—C12—S2—C15	−1.6 (3)
N1—C1—C2—C12	−165.9 (4)	C13—C14—C15—S2	−1.2 (6)
N1—C1—C8—C9	−169.2 (4)	C14—C15—S2—C12	1.6 (4)
N1—C1—C8—S1	5.6 (4)	S2—C12—C13—C14	1.2 (5)
N2—C2—C12—C13	−126.6 (4)	C16—N4—C22—C18	1.6 (5)
N2—C2—C12—S2	43.3 (5)	C16—N4—C22—C21	−179.1 (4)
N2—C3—C4—N3	172.7 (4)	C16—C17—C27—C28	124.1 (18)
N2—C3—C7—N1	7.8 (6)	C16—C17—C27—S4	−71.1 (12)
N2—C3—C7—C6	−172.9 (4)	C16—C17—C27B—C28B	−68 (3)
N3—C5—C6—C7	0.3 (7)	C16—C17—C27B—S4B	108 (3)
N4—C16—C17—N5	−5.2 (5)	C16—C23—C24—C25	−177.9 (4)
N4—C16—C17—C27	175.2 (7)	C16—C23—S3—C26	178.9 (3)
N4—C16—C17—C27B	166.7 (14)	C17—N5—C18—C19	177.9 (4)
N4—C16—C23—C24	170.4 (4)	C17—N5—C18—C22	1.6 (6)
N4—C16—C23—S3	−7.4 (4)	C17—C16—C23—C24	−8.5 (6)
N5—C17—C27—C28	−55 (2)	C17—C16—C23—S3	173.7 (3)
N5—C17—C27—S4	109.3 (11)	C17—C27—C28—C29	174.9 (14)
N5—C17—C27B—C28B	104 (3)	C17—C27—S4—C30	−173.6 (13)
N5—C17—C27B—S4B	−80 (3)	C17—C27B—C28B—C29B	173 (3)
N5—C18—C19—N6	−176.8 (4)	C17—C27B—S4B—C30B	−176 (3)
N5—C18—C22—N4	−4.0 (6)	C18—N5—C17—C16	2.8 (5)
N5—C18—C22—C21	176.7 (4)	C18—N5—C17—C27	−177.6 (6)
N6—C20—C21—C22	−0.6 (7)	C18—N5—C17—C27B	−170.2 (12)
C1—N1—C7—C3	−5.5 (5)	C19—N6—C20—C21	0.7 (7)
C1—N1—C7—C6	175.3 (4)	C19—C18—C22—N4	179.8 (4)
C1—C2—C12—C13	51.8 (6)	C19—C18—C22—C21	0.5 (6)
C1—C2—C12—S2	−138.3 (3)	C20—N6—C19—C18	−0.1 (6)
C1—C8—C9—C10	175.5 (4)	C20—C21—C22—N4	−179.2 (4)
C1—C8—S1—C11	−176.6 (3)	C20—C21—C22—C18	0.0 (6)
C2—N2—C3—C4	−175.5 (3)	C22—N4—C16—C17	2.7 (5)
C2—N2—C3—C7	0.4 (5)	C22—N4—C16—C23	−176.1 (3)
C2—C1—C8—C9	9.0 (7)	C22—C18—C19—N6	−0.4 (6)
C2—C1—C8—S1	−176.2 (3)	C23—C16—C17—N5	173.6 (3)
C2—C12—C13—C14	172.0 (4)	C23—C16—C17—C27	−6.0 (8)
C2—C12—S2—C15	−173.1 (3)	C23—C16—C17—C27B	−14.5 (15)
C3—N2—C2—C1	−10.0 (5)	C23—C24—C25—C26	−0.9 (6)
C3—N2—C2—C12	168.5 (3)	C24—C23—S3—C26	0.7 (3)
C4—N3—C5—C6	−0.4 (7)	C24—C25—C26—S3	1.4 (6)
C4—C3—C7—N1	−176.3 (3)	C25—C26—S3—C23	−1.2 (4)
C4—C3—C7—C6	3.0 (6)	S3—C23—C24—C25	0.0 (4)
C5—N3—C4—C3	1.9 (6)	C27—C28—C29—C30	−7 (2)
C5—C6—C7—N1	177.7 (4)	C28—C27—S4—C30	−6.8 (15)
C5—C6—C7—C3	−1.6 (6)	C28—C29—C30—S4	1.4 (18)
C7—N1—C1—C2	−4.0 (5)	C29—C30—S4—C27	3.1 (10)
C7—N1—C1—C8	174.2 (3)	S4—C27—C28—C29	9 (2)
C7—C3—C4—N3	−3.2 (6)	C27B—C28B—C29B—C30B	5 (2)
C8—C1—C2—N2	−165.6 (3)	C28B—C27B—S4B—C30B	1 (3)
C8—C1—C2—C12	16.0 (6)	C28B—C29B—C30B—S4B	−4 (3)
C8—C9—C10—C11	0.2 (7)	C29B—C30B—S4B—C27B	2 (3)
C9—C8—S1—C11	−0.8 (3)	S4B—C27B—C28B—C29B	−3 (2)

### Hydrogen-bond geometry (Å, º)

**Table:**

*D*—H···*A*	*D*—H	H···*A*	*D*···*A*	*D*—H···*A*
C6—H6···S4^i^	0.93	2.92	3.665 (6)	138
C9—H9···O2^ii^	0.93	2.64	3.465 (8)	149
C11—H11···O5^i^	0.93	2.65	3.520 (8)	156
C14—H14···N5^iii^	0.93	2.69	3.393 (5)	133
C24—H24···S4	0.93	2.77	3.321 (6)	119
C24—H24···S4*B*	0.93	3.00	3.774 (17)	141
C29—H29···O4^iv^	0.93	2.49	3.330 (12)	150
C30—H30···Cl1^v^	0.93	2.96	3.738 (10)	142
C30—H30···O1^v^	0.93	2.40	3.312 (10)	166
C28*B*—H28*B*···S1^vi^	0.93	2.93	3.67 (2)	138
C29*B*—H29*B*···O2^v^	0.93	2.31	3.21 (2)	162
O6—H6*A*···O5	0.82	1.99	2.805 (8)	175
O5—H5*A*···O3^vii^	0.82	2.11	2.889 (8)	158

Symmetry codes: (i) *x*−1, *y*, *z*+1; (ii) *x*, *y*, *z*+1; (iii) −*x*+1, *y*−1/2, −*z*+1; (iv) −*x*+1, *y*+1/2, −*z*; (v) −*x*+2, *y*+1/2, −*z*; (vi) *x*+1, *y*, *z*−1; (vii) −*x*+2, *y*+1/2, −*z*+1.
